# Establishing Area-Specific Brain Organoids Through Transcription Factor-Mediated Patterning

**DOI:** 10.3390/biology15060488

**Published:** 2026-03-19

**Authors:** Jonghun Kim, Yoon-Sun Jang, Minseong Lee, Na Young Choi, Yooju Jung, Junho Lim, Tae Hwan Kwak

**Affiliations:** 1 Department of Genetics, Yale Stem Cell Center, Yale Child Study Center, Yale School of Medicine, New Haven, CT 06520, USA; 2 Department of Biological Science, Konkuk University, Seoul 05029, Republic of Korea; 3 Bioprinting Laboratories Inc., Dallas, TX 75234, USA; 4 Department of Biomedical Engineering, University of North Texas, Denton, TX 76207, USA; 5 Next&Bio Inc., Seoul 02841, Republic of Korea

**Keywords:** brain organoids, single-cell RNA sequencing, cortical arealization, transcription factors

## Abstract

The human cerebral cortex is organized into distinct area-specific regions along the rostral–caudal axis, yet current human brain organoid models incompletely recapitulate this regional diversity. Here, we establish an area-specific brain organoid platform by leveraging transcription factors (TFs) identified through re-analysis of in vivo human cortical transcriptomic datasets. Publicly available single-cell RNA sequencing datasets from human developing cortex were re-analyzed to identify differentially expressed genes associated with rostral and caudal cortical identities. From this analysis, we identified *SP9* (rostral-enriched) and *DMRTA2* (caudal-enriched) as candidate TFs governing regional specification. To model cortical area identity, these TFs were overexpressed in an inducible manner during human cerebral organoid (hCO) generation. Overexpression of *SP9* resulted in hCOs exhibiting rostral cortical characteristics, whereas *DMRTA2* overexpression promoted caudal cortical features. The resulting hCOs showed distinct regional identities, reflected by differential expression of area-specific markers. In addition, these regional identities were accompanied by distinct functional phenotypes, as calcium imaging revealed divergent patterns of spontaneous neural activity between rostral and caudal hCOs. Altogether, our findings demonstrate that overexpression of TFs enables the controlled generation of area-specific hCOs. This approach provides a scalable and reproducible platform for studying human cortical regionalization and offers a framework for investigating region-specific mechanisms underlying neurodevelopmental and neurological disorders.

## Simple Summary

The human cerebral cortex is organized into distinct regions with specialized functions, but current brain organoid models have limited ability to reproducibly generate region-specific identities. In this study, we used human single-cell RNA sequencing data to identify transcription factors enriched in specific cortical regions and show that overexpression of these factors is sufficient to bias human cerebral organoids toward rostral or caudal cortical identities without disrupting overall neural development. This transcription-factor-based approach provides a simple and scalable strategy for generating regionally specified brain organoids and may enhance studies of region-specific brain development and neurological disease mechanisms.

## 1. Introduction

The human cerebral cortex exhibits pronounced regional specialization across multiple spatial axes, such as anterior–posterior, medial–lateral, and dorsal–ventral dimensions [1]. These patterns are established during early neurodevelopment through coordinated transcriptional programming and morphogen signaling. Among these, patterning along the rostral–caudal axis plays a key role in defining cortical areal identity and functional specialization, giving rise to distinct regions such as the frontal and visual cortices [2,3,4]. Disruption of these processes during neurodevelopment has been implicated in several neurodevelopmental and neurological disorders [5,6], highlighting the importance of understanding cortical regionalization in the human brain.

Recent advances in single-cell RNA sequencing (scRNA-seq) technologies have enabled high-resolution mapping of gene expression across developing and adult human cortical areas, revealing distinct area-specific transcriptional signatures associated with regional identity, maturation, and function [7,8,9,10]. These studies have identified various transcription factors (TFs) and regional marker genes that are differentially expressed across distinct cortical regions, providing new insights into the molecular mechanisms underlying human cortical arealization. Despite these advances, translating area-specific transcriptional signatures into experimental model systems that faithfully recapitulate human cortical regional identity remains a major challenge.

Human brain organoids have emerged as a powerful tool for studying human cortical development and disease in vitro [11,12,13,14]. These recapitulate key features of early corticogenesis, including neural progenitor expansion and neuronal differentiation. However, current cerebral organoid (hCO) protocols largely lack controlled regional specification and mostly generate heterogeneous tissues that do not reliably represent distinct cortical areas along the rostral–caudal axis. This limitation makes it difficult to study region-specific development and disease phenotypes in vitro. Previous studies have demonstrated that various morphogens such as FGF8 can anteriorize human brain organoids and promote rostral cortical identities [15,16]. However, morphogen-based approaches require precise control of signaling gradient and may lead to batch-to-batch variation.

To overcome these, in this study, we established an area-specific hCO platform by TF-based patterning. Using publicly available in vivo human cortical scRNA-seq datasets, we identified *SP9* and *DMRTA2* as candidate TFs enriched in rostral and caudal cortical regions, respectively. Inducible overexpression of these TFs during hCO generation enabled the controlled induction of rostral and caudal cortical identities, as assessed by area-specific marker expression. In addition, rostral and caudal hCOs exhibited distinct patterns of neural activity, consistent with region-specific functional difference. This platform provides a scalable and reproducible approach for modeling human cortical regionalization and offers a foundation for studying region-specific mechanisms underlying neurodevelopmental and neurological disorders.

## 2. Materials and Methods

### 2.1. Generation of Area-Specific Cerebral Organoids

To establish the DOX-inducible system, H9 human embryonic stem cells (hESCs) were transduced with lentiviral vectors encoding *SP9* or *DMRTA2*, using the pLVX-TetOne-Puro vector as the lentiviral backbone. Two days post-transduction, puromycin (1 µg/mL) was applied for 3 days for selection. Following selection, hESCs were expanded for an additional 5 days in mTeSR1 medium (STEMCELL Technologies, Vancouver, BC, Canada).

To generate hCOs, hESCs were differentiated following previously established protocols [12,17]. Briefly, cells were dissociated into single cells using TrypLE (Gibco, Waltham, MA, USA) and seeded into ultra-low-attachment 96-well plates at a density of 9000 cells per well in EB formation medium (EBM) supplemented with 50 μM Y-27632 (Calbiochem, San Diego, CA, USA). EBM consisted of DMEM/F12 (Corning, Corning, NY, USA) supplemented with 20% KSR (Gibco), 1% penicillin/streptomycin (P/S) (Gibco), 1% GlutaMAX (Gibco), 1% NEAA (Gibco), 55 μM β-mercaptoethanol (Gibco), 3% FBS (Seradigm, Radnor, PA, USA), and 4 ng/mL bFGF (Peprotech, London, UK). After 5 days, the culture medium was replaced with neural induction medium (NIM), consisting of DMEM/F12 (Corning) supplemented with N2 supplement (Gibco), 1% NEAA (Gibco), 1% GlutaMAX (Gibco), and 1 μg/mL heparin (Sigma, St. Louis, MO, USA). On day 7, organoids were embedded into growth-factor-reduced Matrigel (Corning) droplets and subsequently cultured in neural expansion medium (NEM). NEM consisted of a 1:1 mixture of DMEM/F12 (Corning) and neurobasal medium (Gibco) supplemented with 0.5% NEAA (Gibco), 1% GlutaMAX (Gibco), 1% P/S (Gibco), β-mercaptoethanol (Gibco), 10 μg/mL insulin, N2 supplement (Gibco), and B27 supplement without vitamin A (Gibco). An amount of 2 µg/mL DOX was added from day 5 to day 10 to induce TF expression. On day 10, organoids were cultured on an orbital shaker with neural differentiation medium (NDD). NDD consisted of the same basal components as NEM, with the addition of B27 supplement. Culture medium was changed every 3 days.

### 2.2. Real-Time Quantitative PCR

Total RNA was isolated using the RNeasy Mini Kit (Qiagen, Germantown, MD, USA). An amount of 1 µg of total RNA was reverse-transcribed into cDNA using the High-Capacity cDNA Reverse Transcription Kit (Applied Biosystems, San Francisco, CA, USA). Real-time quantitative PCR (qPCR) was performed with the SYBR Green PCR Master Mix (Applied Biosystems) using the ABI 7500 real-time PCR system (Applied Biosystems). The primer sequences used in this study are listed in Table 1.

### 2.3. Organoid Sample Preparation and Immunohistochemistry

Organoids were fixed in 4% paraformaldehyde (PFA) at 4 °C overnight. After three washes with PBS, the fixed organoids were incubated in 30% sucrose solution at 4 °C for 2 days for cryoprotection. The samples were embedded in OCT compound, cryosectioned at 40 µm using a cryostat (Leica Biosystems, Nussloch, Germany), and mounted onto glass slides. For immunohistochemistry, sections were washed with PBS to remove residual OCT and blocked in PBS containing 10% FBS and 0.5% Triton X-100 for 1 h at RT. The sections were then incubated overnight at 4 °C with the appropriate primary antibodies. The primary antibodies were washed three times with PBS, and then the sections were incubated with the appropriate secondary antibodies containing 10% FBS and 0.5% Triton X-100 for 1 h. Nuclei were counterstained with DAPI. Primary antibodies used in this study were as follows: SOX2 (R&D system, Minneapolis, MN, USA), TBR1 (Abcam, Cambridge, MA, USA), FOXG1 (Abcam), MAP2 (Merk Millipore, Burlington, MA, USA), AUTS2 (Abcam), and NR2F1 (Novus Biologicals, Centennial, CO, USA).

For quantification, DAPI was used to identify nuclei, and positivity was scored at the single-cell level based on nuclear localization and signal-to-background. Specifically, nuclei were classified as positive when FOXG1, AUTS2, or NR2F1 signal showed clear nuclear localization overlapping with DAPI and was visibly above local background. Positive cells were expressed as the percentage of marker-positive nuclei among total DAPI-positive nuclei within each section. To minimize subjectivity, counts were manually double-checked by an independent scorer blinded to the experimental conditions.

### 2.4. Single-Cell RNA Sequencing Data Analysis

Publicly available scRNA-seq data of the developing human cortex were obtained from the cortex-dev dataset hosted on the UCSC Cell Browser. This dataset is derived from previously published scRNA-seq experiments performed on microdissected regions of the human fetal telencephalon [10]. The samples are largely composed of cells from mid-fetal developmental stages.

The gene-by-cell expression matrix (exprMatrix.tsv.gz) and corresponding cell metadata (meta.tsv) were downloaded directly from the UCSC Cell Browser repository and analyzed in R (v4.4.1) using the Seurat (v4) package. Cells were filtered to retain those with 200–6000 detected genes. When mitochondrial gene expression information was available, cells with ≥15% mitochondrial transcript content were excluded from further analysis. Highly variable genes were identified using the variance-stabilizing transformation (VST) method, selecting 3000 features, followed by data scaling. Dimensionality reduction was performed using principal component analysis (PCA), and the top principal components were used to construct a shared nearest-neighbor graph. Cells were clustered using a graph-based clustering approach (resolution = 0.4) and visualized using Uniform Manifold Approximation and Projection (UMAP). Cluster-enriched marker genes were identified using Seurat’s FindAllMarkers function with a Wilcoxon rank-sum test, considering only positively enriched genes (min.pct = 0.25; log2 fold-change threshold = 0.25). Cell clusters were annotated into major neural and non-neural cell types based on canonical marker genes, including *NEUROD6* for excitatory neurons, *PAX6* for radial glial cells, *GAD1* for inhibitory neurons, *COL1A1* for mesenchymal cells, *OLIG1* for oligodendrocytes, *IBA1* for microglia, and VE-cadherin for endothelial cells.

For regional comparisons, cells annotated as PFC-like or V1-like based on metadata information were subset, and DEG analysis was performed using FindMarkers. DEGs were ranked by adjusted *p*-values and visualized using dot plots.

### 2.5. Calcium Imaging

Organoids were transduced with AAV-GCaMP6s.WPRE.SV40 (Addgene, #100843, Watertown, MA, USA). After two weeks of viral infection, intact organoids were used for calcium imaging. Time-lapse images were acquired at a frame rate of 5 frames/s.

### 2.6. Statistical Analysis

Data are presented as mean ± standard deviation (SD). Statistical significance was determined using two-tailed *t*-tests or ANOVA (N.S., not significant; * *p* < 0.05, ** *p* < 0.01, and *** *p* < 0.001).

## 3. Results

### 3.1. Identification of Rostral- and Caudal-Specific Transcription Factors via Single-Cell RNA Sequencing

**Figure 1:**
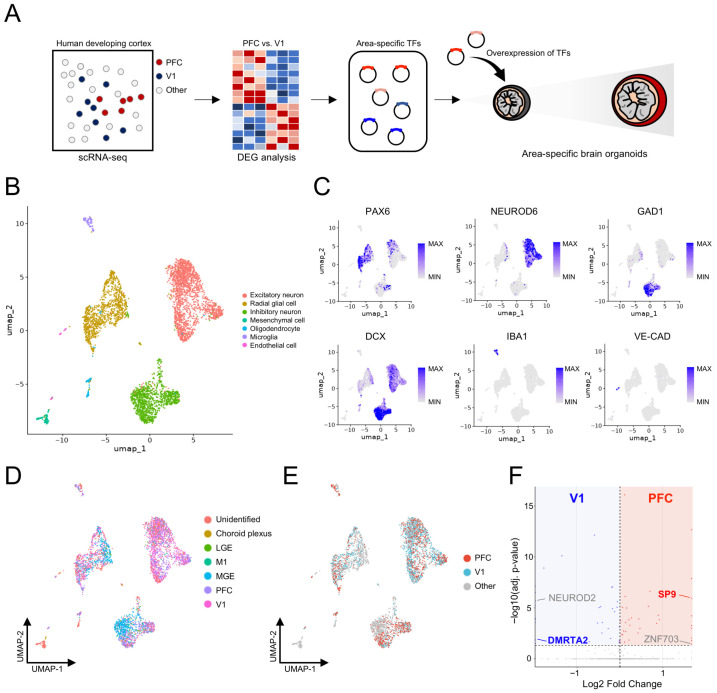
Identification of transcription factors enriched in rostral and caudal cortical regions using transcriptomic data. (**A**). Schematic illustration showing the overall experimental workflow. (**B**). UMAP visualization of scRNA-seq data showing distinct cell types in the developing human cortex. (**C**). UMAP plots displaying the expression of representative cell-type-specific markers, including *PAX6* (radial glial cells), *NEUROD6* (excitatory neurons), *GAD1* (inhibitory neurons), *DCX* (neurons), *IBA1* (microglia), and *VE-CAD* (endothelial cells). (**D**). UMAP visualization showing annotation of cells by cortical region of origin, including PFC, M1, V1, MGE, LGE, and choroid plexus. (**E**). UMAP highlighting cells derived from PFC (red) and V1 (blue). (**F**). DEG analysis between PFC and V1 identifying region-specific TFs. Red-shaded regions indicate PFC-enriched genes, whereas blue-shaded regions indicate V1-enriched genes. Only TFs are annotated. scRNA-seq, single-cell RNA sequencing; PFC, prefrontal cortex; M1, primary motor cortex; V1, primary visual cortex; MGE, medial ganglionic eminence; LGE, lateral ganglionic eminence; DEG, differentially expressed gene; TFs, transcription factors.

To identify area-specific TFs, we first analyzed previously published human fetal brain scRNA-seq datasets [10] in which anatomically defined cortical areas, including the frontal cortex (rostral region) and visual cortex (caudal region), were independently dissected and profiled (Figure 1A). To establish a comprehensive cellular landscape of the developing human cortex, all cells were subjected to unsupervised clustering based on their transcriptional profiles. This analysis revealed major neural cell types, including radial glial cells, excitatory neurons, and inhibitory neurons, as well as non-neural cell populations, such as microglia and endothelial cells (Figure 1B). Each cell cluster exhibited cell-type-specific marker gene expression, including *PAX6* for radial glial cells, *NEUROD6* for excitatory neurons, *GAD1* for inhibitory neurons, *IBA1* for microglia, and *VE-cadherin* for endothelial cells (Figure 1C). This annotation confirmed that both neural and non-neural populations were robustly captured in the dataset.

Next, cells were categorized according to their cortical area of origin, including the prefrontal cortex (PFC), primary motor cortex (M1), primary visual cortex (V1), medial ganglionic eminence (MGE), lateral ganglionic eminence (LGE), and choroid plexus (Figure 1D). Subsequently, we focused our analysis on two well-defined cortical regions representing these domains: PFC as the rostral region and V1 as the caudal region (Figure 1E). Differentially expressed gene (DEG) analysis was then performed between PFC and V1 to identify region-specific TFs (Figure 1F). We identified 35 and 31 genes that were significantly upregulated in the PFC-like and V1-like regions, respectively (*p*-value < 0.05). Among these genes, *SP9* and *ZNF703* were identified as TFs preferentially upregulated in the PFC, whereas *NEUROD2* and *DMRTA2* were identified as TFs enriched in the V1. Although many DEGs showed stronger statistical significance, we prioritized TF candidates with robust regional enrichment and clear biological relevance to rostral–caudal telencephalic patterning, as these are best suited for functional perturbation. Based on these, *SP9* and *DMRTA2* were selected for further functional validation as representative rostral- and caudal-enriched TFs, respectively (*SP9*: log2FC = 1.64 in PFC vs. V1; *DMRTA2*: log2FC = 1.49 in V1 vs. PFC). *SP9* showed robust enrichment in the PFC in our scRNA-seq analyses and belongs to the Sp-family of TFs. *SP8* and *SP9* have been shown to function in a partially redundant and cooperative manner during early vertebrate development and are associated with FGF signaling pathways, as *Sp8*/*Sp9* regulate *Fgf8* expression during embryogenesis [18], and *Sp8* exhibits reciprocal induction with *Fgf8* [19], a key anterior morphogen that governs rostral/caudal patterning of the developing telencephalon [20]. In contrast, *DMRTA2* was strongly enriched in the V1 in our scRNA-seq analyses and has been reported to exhibit a graded expression pattern in the developing telencephalon, with a rostral-low/caudal-high gradient [21].

### 3.2. Generation and Characterization of Rostral and Caudal Human Cerebral Organoids

**Figure 2:**
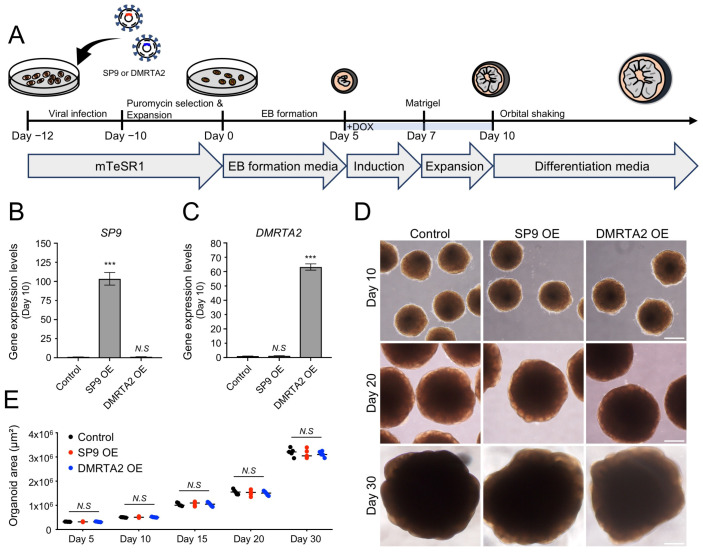
Generation of region-specific human cerebral organoids using a DOX-inducible system. (**A**). Schematic illustration showing the strategy for generating region-specific hCOs. (**B**). Expression levels of SP9 at day 10 in control, SP9-overexpressing, and DMRTA2-overexpressing hCOs. Data are shown as the mean ± SD (*n* = 3 biological replicates). (**C**). Expression levels of DMRTA2 at day 10 in control, SP9-overexpressing, and DMRTA2-overexpressing hCOs. Data are shown as the mean ± SD (*n* = 3 biological replicates). (**D**). Bright-field images of hCOs at days 10, 20, and 30. Scale bar, 500 μm. (**E**). Quantification of organoid size during hCO generation. Data are shown as the mean ± SD (*n* = 5 organoids from two independent batches). Statistical significance was determined using two-tailed *t*-tests (**B**,**C**) or ANOVA (**E**). N.S, not significant; *** *p* < 0.001. hCOs, human cerebral organoids.

To investigate whether the identified TFs are sufficient for cortical area identity, we next established a doxycycline (DOX)-inducible expression system to overexpress *SP9* or *DMRTA2* during hCO generation (Figure 2A). Human embryonic stem cells (hESCs) harboring DOX-inducible *SP9* or *DMRTA2* were differentiated into hCOs using established protocols [12]. For transient TF expression, DOX was applied for 5 days (from day 5 to day 10) during the induction and expansion stages.

First, we confirmed robust induction of *SP9* in SP9-overexpressing hCOs (103.4 ± 8.2-fold relative to control; Figure 2B), whereas *DMRTA2* was significantly upregulated in DMRTA2-overexpressing hCOs (63.2 ± 2.2-fold relative to control) at day 10 (Figure 2C). Notably, the overexpression levels exceed the endogenous area-associated fold-changes observed in the scRNA-seq data, consistent with a gain-of-function approach designed to test sufficiency rather than to mimic physiological effect sizes. All experimental conditions, including control, SP9-overexpressing, and DMRTA2-overexpressing hCOs, exhibited comparable and robust differentiation, with similar morphology and comparable organoid size (control: 3.19 ± 0.17 × 10^6^ µm^2^; SP9-overexpressing: 3.13 ± 0.18 × 10^6^ µm^2^; DMRTA2-overexpressing: 3.12 ± 0.11 × 10^6^ µm^2^, day 30; Figure 2D,E). These results indicate that transient TF overexpression did not impair overall hCO development.

**Figure 3:**
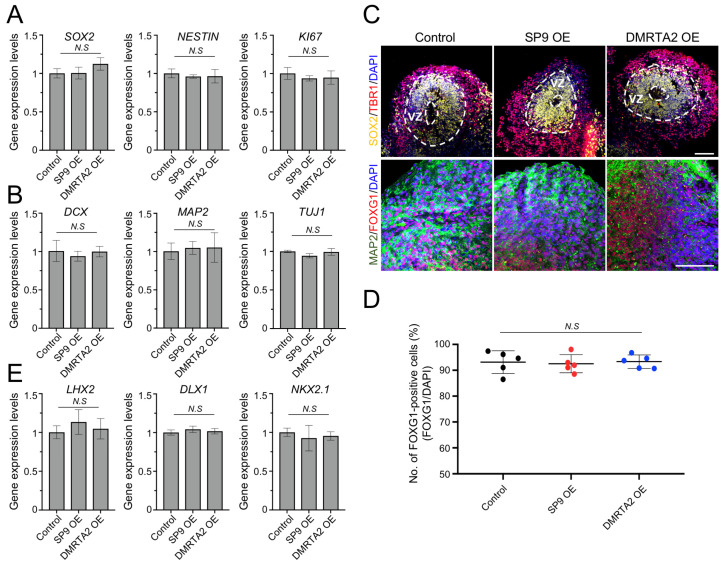
Characterization of region-specific human cerebral organoids at day 35. (**A**). Expression levels of radial glial cell markers (*SOX2*, *NESTIN*, *KI67*). Data are shown as the mean ± SD (*n* = 3 biological replicates). (**B**). Expression levels of neural markers (*DCX*, *MAP2*, *TUJ1*). Data are shown as the mean ± SD (*n* = 3 biological replicates). (**C**). Representative immunofluorescence images of hCOs at day 30. Upper panel: SOX2 staining with the neuronal marker TBR1; dotted circles indicate VZ-like regions. Lower panel: FOXG1 staining with the neuronal marker MAP2. Scale bars, 100 μm. (**D**). Quantification of FOXG1-positive cells in control, SP9-overexpressing, and DMRTA2-overexpressing hCOs. Data are shown as the mean ± SD (*n* = 5 organoids from two independent batches). (**E**). Expression levels of dorsal–ventral markers (*LHX2*, *DLX1*, *NKX2.1*). Data are shown as mean ± SD (*n* = 3 biological replicates). Statistical significance was determined by ANOVA (**A**,**B**,**D**,**E**). N.S, not significant. hCOs, human cerebral organoids. VZ, ventricular zone.

We next examined whether hCO development was comparable across control, SP9-overexpressing, and DMRTA2-overexpressing hCOs. The expression levels of typical radial glial cell markers, including *SOX2*, *NESTIN*, and KI67, were comparable among the three groups (Figure 3A). In addition, the expression of neuronal markers such as *DCX*, *MAP2*, and *TUJ1* was similar across all conditions (Figure 3B), further confirming comparable neural differentiation. Moreover, all conditions formed ventricular zone (VZ)-like structures, characterized by SOX2-positive domains, and the proportion of telencephalic marker FOXG1-positive cells was comparable across groups (control: 93.1 ± 4.4%; SP9-overexpressing: 92.5 ± 3.5%; DMRTA2-overexpressing: 93.3 ± 2.6%; Figure 3C,D), indicating preserved forebrain identity. Notably, the expression of dorsal–ventral patterning markers such as *LHX2*, *DLX1*, and *NKX2.1* was comparable across all conditions (Figure 3E), indicating that transient TF overexpression did not perturb dorsal–ventral axis specification.

Collectively, these data suggest that transient cortical area-specific TF overexpression does not affect overall hCO development or forebrain identity.

### 3.3. Distinct Molecular Features of Rostral and Caudal Human Cerebral Organoids

**Figure 4:**
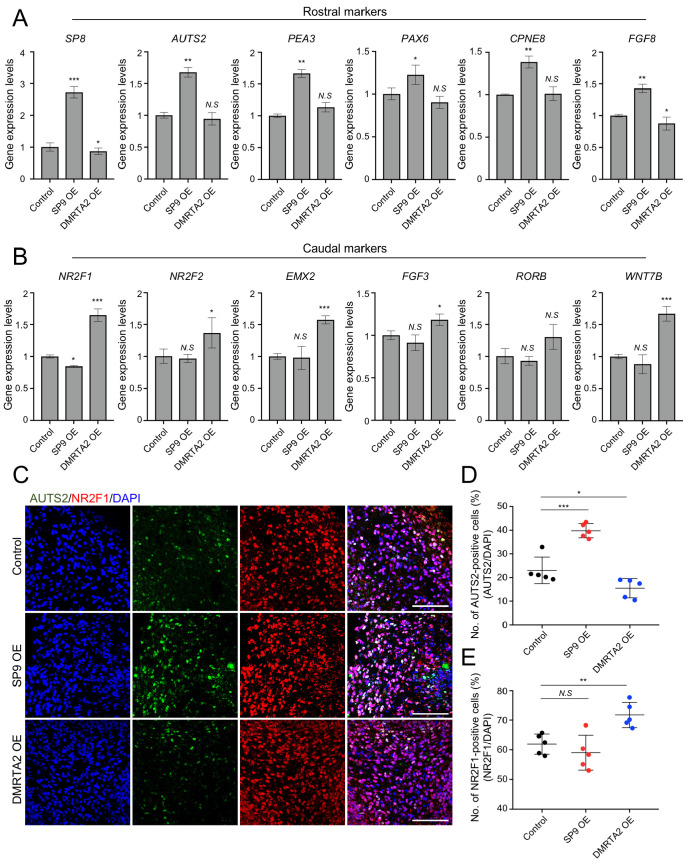
Distinct molecular signatures of rostral and caudal human cerebral organoids. (**A**). Expression levels of rostral markers (*SP8*, *AUTS2*, *PEA3*, *PAX6*, *CPNE8*, *FGF8*). Data are shown as the mean ± SD (*n* = 3 biological replicates). (**B**). Expression levels of caudal markers (*NR2F1*, *NR2F2*, *EMX2*, *FGF3*, *RORB*, *WNT7B*). Data are shown as the mean ± SD (*n* = 3 biological replicates). (**C**). Representative immunofluorescence images of the rostral marker AUTS2 and the caudal marker NR2F1 in hCOs at day 30. Scale bars, 100 μm. (**D**). Quantification of AUTS2-positive cells in control, SP9-overexpressing, and DMRTA2-overexpressing hCOs Data are shown as the mean ± SD (*n* = 5 organoids from two independent batches). (**E**). Quantification of NR2F1-positive cells in control, SP9-overexpressing, and DMRTA2-overexpressing hCOs. Data are shown as the mean ± SD (*n* = 5 organoids from two independent batches). Statistical significance was determined using two-tailed *t*-tests (**A**,**B**,**D**,**E**). N.S, not significant; * *p* < 0.05, ** *p* < 0.01, and *** *p* < 0.001.

To determine whether transient overexpression of *SP9* or *DMRTA2* induces region-specific molecular identities, we next examined gene expression profiles in hCOs. Rostral markers, including *SP8*, *AUTS2*, *PEA3*, *PAX6*, and *CPNE8*, were significantly upregulated in SP9-overexpressing hCOs compared with control and DMRTA2-overexpressing hCOs. Notably, expression of *FGF8*, a key regulator of rostral cortical patterning, was significantly increased in SP9-overexpressing hCOs, further supporting the acquisition of a rostral cortical molecular identity (Figure 4A). In contrast, DMRTA2-overexpressing hCOs showed significantly increased expression of caudal markers, including *NR2F1*, *NR2F2*, *EMX2*, and *FGF3*, compared with control and SP9-overexpressing hCOs; *RORB* showed an upward trend but did not reach statistical significance. These findings support a caudal cortical molecular identity. Furthermore, a WNT signaling-related gene, *WNT7B*, was upregulated in DMRTA2-overexpressing hCOs, consistent with enhanced posterior/caudal cortical patterning (Figure 4B).

To further validate these region-specific molecular changes at the cellular level, we next performed immunostaining for AUTS2 and NR2F1, the representative rostral and caudal markers, respectively (Figure 4C). Consistent with the gene expression analyses, SP9-overexpressing hCOs exhibited a significantly increased proportion of AUTS2-positive cells compared with control and DMRTA2-overexpressing hCOs (SP9-overexpressing: 39.8 ± 2.9% vs control: 23.0 ± 5.3%; DMRTA2 overexpressing: 15.5 ± 4.1%; Figure 4D). In contrast, DMRTA2-overexpressing hCOs showed a significantly increased proportion of NR2F1-positive cells (71.8 ± 4.3%) compared with control (61.9 ± 3.3%) and SP9-overexpressing hCOs (59.8 ± 5.9%; Figure 4E), confirming region-specific cellular patterning induced by TF overexpression. These findings were reproducible across two independent differentiation batches.

Altogether, these data suggest that transient overexpression of *SP9* or *DMRTA2* promotes region-specific molecular and cellular features consistent with rostral or caudal cortical identities in hCOs.

### 3.4. Functional Divergence of Rostral and Caudal Human Cerebral Organoids

**Figure 5:**
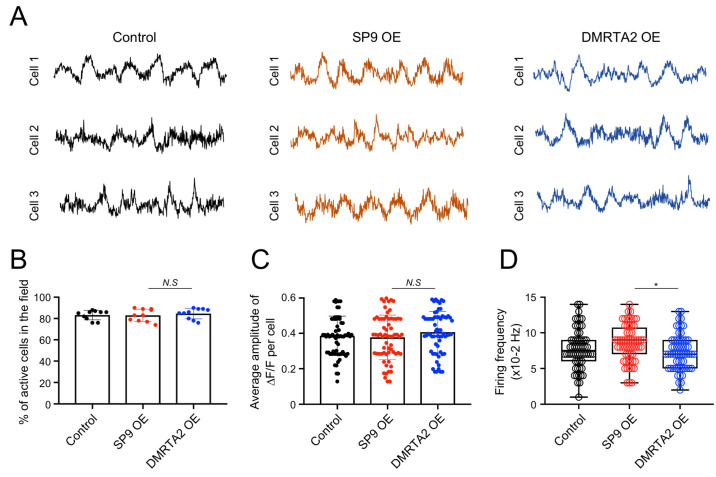
Distinct functional properties of rostral and caudal human cerebral organoids. (**A**). Representative single-cell traces of spontaneous calcium transients in individual neurons. (**B**). Proportion of active cells across control, SP9-overexpressing, and DMRTA2-overexpressing hCOs. Data are shown as the mean ± SD (*n* = 10 fields from 3 organoids per group). (**C**). Quantification of the mean ΔF/F amplitude per cell in hCOs (*n* = 65 cells from 3 organoids per group). (**D**). Quantification of calcium transient frequency per cell in hCOs (*n* = 60 cells from 3 organoids per group). Statistical significance was determined using two-tailed *t*-tests (B–D). N.S, not significant; * *p* < 0.05.

Lastly, given that rostral and caudal cortical regions are known to exhibit distinct patterns of neuronal activity during development [22], we next asked whether the molecular differences induced by *SP9* or *DMRTA2* overexpression are accompanied by functional changes. To address this, we assessed spontaneous neural activity in hCOs at day 50 using calcium imaging. To monitor neuronal calcium dynamics, hCOs were transduced with a lentiviral vector encoding the calcium indicator GCaMP6s. Calcium activity patterns were then compared among control, SP9-overexpressing, and DMRTA2-overexpressing hCOs (Figure 5A).

Calcium imaging revealed that the overall proportion of active cells was comparable among control, SP9-overexpressing, and DMRTA2-overexpressing hCOs (control: 83.2 ± 4.5%; SP9-overexpressing: 83.0 ± 5.8%; DMRTA2-overexpressing: 84.6 ± 5.0%; Figure 5B), indicating that TF overexpression did not alter the fraction of neurons exhibiting spontaneous calcium activity. In addition, the amplitude of calcium transients, measured as peak ΔF/F, did not differ significantly across conditions (Figure 5C), suggesting that the magnitude of individual calcium events was largely preserved. In contrast, the frequency of spontaneous calcium transients differed between SP9-overexpressing and DMRTA2-overexpressing hCOs (SP9-overexpressing: 8.53 ± 2.60 events per recording vs DMRTA2-overexpressing: 7.33 ± 2.50 events per recording; Figure 5D), reflecting differences in the temporal dynamics of neuronal activity.

Overall, these data suggest that transient overexpression of *SP9* or *DMRTA2* is associated with distinct temporal patterns of spontaneous neuronal activity in hCOs, without altering the overall proportion of active cells or the amplitude of calcium transients.

## 4. Discussion

Human cortical regionalization emerges through tightly coordinated transcriptional programs operating across multiple spatial axes during development [23]. While human brain organoid models have provided powerful platforms for modeling early corticogenesis, it remains difficult to reliably generate organoids with defined cortical area identities. In this study, we demonstrate that transient and inducible overexpression of area-specific TFs is sufficient to bias hCOs toward rostral or caudal cortical identities, offering a complementary strategy to existing morphogen-based approaches.

Recent advances in scRNA-seq have provided unprecedented resolution for dissecting transcriptional heterogeneity across the developing human cortex, enabling systematic identification of genes and regulatory programs associated with specific cortical areas [7,8,9,10]. Unlike bulk RNA-seq, scRNA-seq provides the cellular context needed to interpret regional transcriptional signatures in the presence of differences in cell-type composition and developmental state. In the current study, while the PFC-V1 DEG comparison was performed in an aggregate (bulk-like) manner across all captured cells, scRNA-seq remains advantageous because this aggregation is performed over single-cell-resolved data. This enables annotation of major cell types/states in each anatomically defined region and provides critical quality control to evaluate potential contributions from cell-type composition, maturation state, or mixed-identity/boundary populations. Based on this framework, we were able to rationally select area-enriched TFs as candidate regulators of cortical areal identity. This data-driven approach enables the translation of descriptive human scRNA-seq resources into experimentally testable perturbations in brain organoids, providing a principled strategy for imposing region-specific identities in vitro.

Importantly, our findings indicate that cortical area identity can be modulated independently of global neural differentiation programs. Transient induction of *SP9* or *DMRTA2* did not disrupt organoid growth, forebrain specification, or dorsal–ventral patterning, suggesting that cortical areal identity represents a separable developmental layer that can be selectively tuned through transcriptional control. This finding is consistent with developmental models in which early patterning cues establish a permissive cortical field, followed by TF-driven refinement of areal fate [3].

The TF-based strategy described in this study differs fundamentally from morphogen-driven patterning paradigms. Previous studies have demonstrated that recapitulating morphogen gradients governing central nervous system regionalization during neural tube development can successfully generate region-specific brain organoids, particularly for broad domains such as forebrain, midbrain, and hindbrain [24,25,26,27]. However, these strategies face inherent limitations when applied to the cerebral cortex, a brain structure that shares an overall regional identity while exhibiting highly refined molecular, structural, and functional distinctions across its internal cortical areas. In particular, precise control of spatial and temporal morphogen gradients within three-dimensional organoid systems is technically challenging, and even subtle variations in culture conditions can result in substantial differences in patterning outcomes [15,16]. This sensitivity increases batch-to-batch variability and poses a significant risk to reproducibility and scalability. In contrast, TF-mediated patterning offers a genetically defined, scalable, and reproducible means of enforcing regional identity, independent of diffusion-based signaling. This feature is particularly advantageous for disease modeling and comparative studies, where consistency across batches and conditions is critical. From a practical standpoint, TF-mediated patterning is straightforward to standardize (without precise gradient engineering) and offers tighter user-defined control over areal identity, resulting in improved reproducibility for cross-batch and cross-line comparisons.

Notably, the acquisition of region-specific molecular identities was accompanied by differences in the temporal dynamics of spontaneous neuronal activity, without changes in overall activity levels or signal amplitude. These frequency differences are in line with known developmental distinctions between rostral and caudal cortical regions, which differ in maturation timing, connectivity, and intrinsic network dynamics [28,29,30]. These observations suggest that transcriptional areal patterning may influence not only molecular identity but also the temporal organization of emerging neuronal networks, even at early developmental stages.

However, several limitations should be acknowledged. This study focuses on early stages of hCOs, and it remains to be determined whether these region-specific identities are maintained during long-term maturation. Because we did not perform longitudinal analyses at later differentiation stages, we cannot assess the stability, potential drift, or further refinement of rostral/caudal programs over time. Future studies incorporating extended maturation and time-resolved, multi-marker validation will be important to establish the durability of these imposed areal identities. Calcium burst frequency is not a canonical marker of forebrain areal identity. Instead, the observed differences likely reflect downstream changes in network maturation and connectivity driven by TF-based rostral/caudal patterning. While calcium imaging revealed differences in activity frequency, further electrophysiological analyses will be required to dissect the underlying circuit properties and synaptic mechanisms. Finally, future studies will be required to assess whether modulation of these TFs affects disease phenotypes in region-specific disease models.

In summary, our study establishes a TF-based framework for generating area-specific hCOs. This strategy broadens the experimental repertoire for studying human cortical development and offers a flexible platform for investigating region-specific mechanisms in both normal and disease states.

## 5. Conclusions

This study demonstrates that overexpression of area-specific TFs is sufficient to direct hCOs toward rostral or caudal cortical identities without compromising overall neural differentiation. This TF-based approach provides a simple and scalable strategy for generating regionally specified hCOs and may facilitate more precise modeling of region-specific neurodevelopmental processes and disease mechanisms.

## Figures and Tables

**Table 1:** Real-time quantitative PCR primer sequences used in this study.

Gene Name	Primers
*SP9*	F: TCGTGTGCAACTGGCTCTTCTG
	R: TGTGTTTGCTCAGGTGGTCGCT
*DMRTA2*	F: CCTACGAGGTCTTCGGTTCAGT
	R: AGGTCAAACTTCTGCAACTTGGC
*SOX2*	F: GCTACAGCATGATGCAGGACCA
	R: TCTGCGAGCTGGTCATGGAGTT
*NESTIN*	F: TCAAGATGTCCCTCAGCCTGGA
	R: AAGCTGAGGGAAGTCTTGGAGC
*KI67*	F: GAAAGAGTGGCAACCTGCCTTC
	R: GCACCAAGTTTTACTACATCTGCC
*DCX*	F: TATGCGCCGAAGCAAGTCTCCA
	R: CATCCAAGGACAGAGGCAGGTA
*MAP2*	F: AGGCTGTAGCAGTCCTGAAAGG
	R: CTTCCTCCACTGTGACAGTCTG
*TUJ1*	F: TCAGCGTCTACTACAACGAGGC
	R: GCCTGAAGAGATGTCCAAAGGC
*LHX2*	F: ACGCCAAGGACTTGAAGCAGCT
	R: TTTCCTGCCGTAAGAGGTTGCG
*DLX1*	F: CATCAGTTCGGTGCAGTCCTAC
	R: CCTTGCCATTGAAGCGCACTTC
*NKX2.1*	F: CAGGACACCATGAGGAACAGCG
	R: GCCATGTTCTTGCTCACGTCCC
*SP8*	F: GAGGCTACAACTCGGATTACTCG
	R: GTAGCACTGGCTTGAAGCCGTC
*AUTS2*	F: CCTCCTCATCACAGCAACTTCC
	R: GAAGGCATTGCCACCAACTGCT
*PEA3*	F: AGGAACAGACGGACTTCGCCTA
	R: CTGGGAATGGTCGCAGAGGTTT
*PAX6*	F: CTGAGGAATCAGAGAAGACAGGC
	R: ATGGAGCCAGATGTGAAGGAGG
*CPNE8*	F: GCAAAACTGCCTCCAGATGGAAG
	R: TCAGACTCCTGTAATAAGCCTCC
*FGF8*	F: GGACACCTTTGGAAGCAGAGTC
	R: CCAGCACAATCTCCGTGAAGAC
*NR2F1*	F: TGCCTCAAAGCCATCGTGCTGT
	R: CAGCAGCAGTTTGCCAAAACGG
*NR2F2*	F: TGCACGTTGACTCAGCCGAGTA
	R: AAGCACACTGAGACTTTTCCTGC
*EMX2*	F: GCTCATCCACCGCTACCGATAT
	R: TTCTCAAAGGCGTGTTCCAGCC
*FGF3*	F: AAGCTCTACTGCGCCACGAAGT
	R: CCACCTCCACTGCCGTTATCTC
*RORB*	F: TGTGCCATCCAGATCACTCACG
	R: GGTTGAAGGCACGGCACATTCT
*WNT7B*	F: AGAAGACCGTCTTCGGGCAAGA
	R: AGTTGCTCAGGTTCCCTTGGCT

## Data Availability

The datasets in this study are available from the corresponding authors upon reasonable request.
